# The P301L tau mutation alters extracellular vesicle biogenesis in astrocyte and contributes to astrocyte‐mediated tau pathology in a human iPSC‐derived model of tauopathies

**DOI:** 10.1002/alz.088991

**Published:** 2025-01-03

**Authors:** Yang You, Sean D Mann, Wenyan Lu, Wayne W. Poon, Seiko Ikezu, Takahisa Kanekiyo, Dennis W. Dickson, Jarosaw Sławek, Zbigniew Wszolek, Tsuneya Ikezu

**Affiliations:** ^1^ Mayo Clinic, Jacksonville, FL USA; ^2^ University of California, Irvine, Irvine, CA USA; ^3^ St. Adalbert Hospital, Gdańsk Poland

## Abstract

**Background:**

Tauopathies are a group of neurodegenerative disorders which are characterized by the accumulation of abnormal tau protein in the brain. However, the mechanistic understanding of pathogenic tau formation and spread within the brain remains elusive. Astrocytes are major immune reactive cells in the brain and have been implicated in exacerbating tau pathology by releasing extracellular vesicles (AEVs) containing pro‐inflammatory cytokines and chemokines upon activation. Our prior investigation revealed a significant association between AEVs and tau pathology development, as well as cognitive function, by analyzing brain‐derived EV proteins from AD patients. In this study, we explore the potential roles of AEVs in tau pathogenesis using a human induced pluripotent stem cell (iPSC) model.

**Method:**

We obtained two male P301L tau mutant iPSC lines from a Polish family with frontotemporal dementia. By including two male control lines, these iPSCs were differentiated into astrocytes (iAs) and characterized by immunocytochemistry and subjected for bulk RNAseq. Bioinformatics analysis was conducted to compare the transcriptome profile between wild‐type (WT) and P301L iAs. EVs from WT and P301L iAs were isolated by ultracentrifugation combined with size exclusion chromatography. Characterization of WT and P301L iAEVs involved nanoparticle tracking analysis, nano‐flow cytometry and super‐resolution microscopy.

**Result:**

We successfully differentiated WT and P301L mutant iPSC lines into astrocytes with >99% purity. P301L iAs displayed distinctive astrocyte reactivity compared to WT cells, with elevated levels of pan‐reactive astrocyte genes (e.g., GFAP, CD44) and decreased expression of neuroprotective A2 astrocyte‐specific genes (e.g., TM4SF1, PTGS2). Additionally, gene enrichment set analysis of RNAseq data revealed dysregulation in the endo‐lysosomal pathway and extracellular matrix in P301L iAs compared to WT iAs. The count of intraluminal vesicles marked by CD9+ were reduced in P301L iAs compared to WT cells. Moreover, we observed a significant increase in the internalization of Tau by P301L iAs compared to WT iAs following incubation with preformed Tau fibrils, resulting in an augmented release of tau‐containing EVs from P301L iAs.

**Conclusion:**

Our findings suggest a potential alteration in EV biogenesis in P301L iAs, potentially contributing to astrocyte‐mediated tau pathology. Future investigations will focus on understanding how AEVs contribute to tau propagation and accumulation.

